# Effects of Feeding Fermented Cassava Leaves on Intestinal Morphology, Cecal Microbiota, and Metabolome in Hybrid Geese

**DOI:** 10.3390/microorganisms13030660

**Published:** 2025-03-14

**Authors:** Mao Li, Tieshan Xu, Xuejuan Zi, Renlong Lv, Lihong Gu

**Affiliations:** 1 Tropical Crops Genetic Resources Institute, Chinese Academy of Tropical Agricultural Sciences, Danzhou 571737, China; limao@catas.cn (M.L.); xutieshan760412@163.com (T.X.); lvrenlong@aliyun.com (R.L.); 2 Key Laboratory of Ministry of Education for Genetics and Germplasm Innovation of Tropical Special Trees and Ornamental Plants, Key Laboratory of Germplasm Resources of Tropical Special Ornamental Plants of Hainan Province, School of Tropical Agriculture and Forestry, Hainan University, Danzhou 571737, China; zixuejuan@163.com; 3 Institute of Animal Science & Veterinary, Hainan Academy of Agricultural Science, Haikou 571700, China

**Keywords:** goose, fermented cassava leaves, intestinal morphology, cecal microbiota, metabolites

## Abstract

In this study, we characterized the effects of a diet supplemented with fermented cassava leaves (FCLs) on growth performance, intestinal morphology, the cecal microbiota, and cecal metabolites in hybrid geese. We found that the FCL diet was beneficial to goose growth performance and also promoted a healthy intestinal morphology, as reflected by better morphology properties of the duodenum, jejunum, ileum, and cecum. Moreover, the FCL diet significantly altered cecal microbial diversity and composition, increasing the diversity and abundance of the beneficial *Bacteroides*. Further, the FCL diet increased the complexity and stability of cecal microbial co-occurrence network interactions as a result of altered topological distributions in the network, such as edges, density, degree, and betweenness. The FCL diet had clear impacts on the composition and abundance of cecal metabolites, with increases in metabolites involved in amino acid biosynthesis, digestion, and absorption, as well as an upregulation of associated metabolic pathways. Based on these benefits to growth performance, intestinal development, and cecal microbe-mediated metabolism in geese, FCLs can be utilized as a reliable feed resource for geese in tropical and subtropical regions.

## 1. Introduction

Goose farming in China is a traditional industry with over 3000 years of history. Chinese goose production accounts for over 90% of production worldwide, making China the largest producer of geese in the world [1]. Corn and soybean meal are the primary raw materials for goose feed, and the large volume of goose production inevitably consumes a large amount of grain [2]. Appropriate reductions in the use of corn and soybean meal would help reduce feeding costs and also promote food security in China. This could be achieved by developing new sources of feed. Previous studies have found that fermented forage can replace concentrated feed to some extent without affecting production performance. Li et al. [2] reported that the supplementation of goose diet with 12% fermented alfalfa had benefits to immunity, antioxidant status, growth performance, and carcass characteristics. Hong et al. [3] revealed that fermented maize stover could be used as a partial substitute for fattening goose feed, and an addition of 15% could effectively maintain its production performance. Wang et al. [4] showed that fermented paper mulberry silage could enhance growth performance, carcass indexes, and the quality of meat. In sum, these studies indicate that the application of fermented forage in goose diets has strong practicality and enormous potential.

Compared with other poultry, geese can utilize fiber-rich forage. Many studies have shown that the intestinal structure of geese is more conducive to the digestion and utilization of fiber in forage, with the type and content of fiber in feed also affecting the intestinal morphology [3,5]. The cecal microbiota of geese also have important functions in digestion. The numerous and diverse microorganisms in the cecum can degrade structural and non-structural carbohydrates in forage, converting them into short-chain volatile fatty acids and other easily absorbable small molecules, providing nutrients and energy to the host [3,6]. Yan et al. [6] demonstrated that fermented feeds could alter the microflora in goose intestines, enhancing intestinal health and growth performance. In addition, Hong et al. [3] found that fermented maize stover could increase the abundance of advantageous microbes like *Victivallis* and *Coprococcus* in the goose gut. The metabolic products of the gut microbiota have direct impacts on their animal hosts, playing important roles in nutrient metabolism and the synthesis of active ingredients [7]. The abundant microorganisms in the ceca of geese inevitably produce large amounts of metabolites, and exploring the abundance and composition of these metabolites can help shed light on the mechanisms by which feeding forage affects animal health [8]. Unfortunately, there have been few studies on the effects of fermented forage on cecal metabolites in geese.

Cassava (*Manihot esculenta* Crantz) is a widely distributed tropical crop. It has value not only as a food crop but also as an important source of feed [9]. The consumption of feed grains in China is enormous, and thus the development and utilization of cassava feed is of great significance. Previous work from our group found that the addition of cassava leaf powder to the feed is beneficial for goose growth performance, slaughter performance, intestinal development, and microbe diversity, with an optimal addition ratio of 5% [10,11,12]. Meanwhile, feeding with cassava leaf silage was found to improve goat rumen microbial metabolism and enhance growth performance [13]. However, the effects of fermented cassava leaf (FCL) diets on rearing performance in geese have yet to be characterized. Therefore, our goal was to explore the effects of fermented cassava leaf diets on growth performance, intestinal morphology, the cecal microbiota, and cecal metabolites in geese.

## 2. Materials and Methods

This experiment was approved by the Animal Care and Use Committee of the Chinese Academy of Tropical Agricultural Sciences (approval number CATAS-20211015-1, 15 October 2021). All surgeries were performed according to the standards of this document to minimize the suffering of the animals.

### 2.1. Experimental Design

In 2021, this experiment was carried out at the Tropical Crops Genetic Resources Institute of the Chinese Academy of Tropical Agricultural Sciences in Danzhou, China. One hundred and sixty 28-day-old male Hainan hybrid geese with roughly equal body weights were split at random into two groups, with eight replicates of 10 geese per replicate. Geese were fed with either a control diet (CON) without fermented cassava leaves or a supplemented fermented cassava leaf diet (FCL) over a six-week period. Table 1 lists the nutritional information and ingredients of both diets used in this experiment. The procedure of fermenting cassava leaves was as follows: Cassava leaves of the variety NO.SC7 were harvested and cut into tiny pieces (about 0.5 cm). A lactic acid bacteria (LAB, *Lactobacillus plantarum*) inoculant was used to ferment the cassava leaves, and the application rate of LAB was 1.0 × 10^5^ colony-forming units (cfu)/g of fresh matter (FM). The cassava leaves and LAB were mixed homogenously, compacted, and stored in large, sealed plastic drums. After 30 days of anaerobic fermentation, the fermented leaves were examined for quality, and well-fermented products were used in the feeding trial. Both feed and water were freely available to the geese throughout the duration of the experiment. During the experiment, the geese were kept indoors under the same environmental conditions, at a temperature of about 25–30 °C, and were immunized according to the normal immunization program.

### 2.2. Measurement of Growth Performance

The body weights of geese were recorded on the first day and last day of the experiment, with the latter referred to as the final body weight (FBW). The average daily gain (ADG) was then determined. The daily feed intake of each group during the six-week experimental period was recorded and reported as the average daily feed intake (ADFI). The feed conversion ratio (FCR), or the ratio of ADFI to ADG, was also determined.

### 2.3. Measurement of Intestinal Morphology

After six weeks, one healthy goose with an approximately average body weight was selected for slaughter from each replicate of each treatment. Eight geese were chosen from each group, for a total of sixteen geese. Goose intestines were quickly removed, and about 2 cm each from the duodenum, jejunum, middle ileum, and cecum were isolated. These were gently washed with physiological saline, dried using filter paper, and fixed in 10% formaldehyde phosphate buffer. Intestinal samples were embedded in paraffin using standard procedures and stained with hematoxylin and eosin. These embedded and stained sections were then imaged using a fluorescence microscope (BX63, Olympus Corporation, Tokyo, Japan). Image ProPlus 6.0 software (Media Cybernetics, Silver Spring, MD, USA) was used to quantify the muscle thickness, fold height, villus height (VH), and crypt depth (CD). VH/CD was calculated as the ratio of villus height to crypt depth.

### 2.4. Determination of Cecal Microbiota

Cecal contents were collected from the aforementioned slaughtered geese. These samples were flash-frozen in liquid nitrogen and stored at −80 °C for subsequent microbial and metabolomic analyses. DNA from cecal microbes was extracted using the HiPure fecal DNA kit (SAGENEA, Guangzhou, China). The primers 341F (5′-CCTACGGGNGGCWGCAG-3′) and 806R (5′-GGACTACHVGGGTATCTAAT-3’) were used to amplify regions V3 to V4 of the microbial 16S rRNA. 16S amplicon sequencing was then performed on the Illumina HiSeq 2500 System (Illumina, Inc., San Diego, CA, USA). FASTP (version 0.18.0) was used to filter out low-quality bases and reads from the raw sequence data. Reads were clustered into operational taxonomic units (OTUs) with a 97% identity threshold using the UPARSE algorithm (version 9.2.64). The UCHIME algorithm was then used to remove all chimeric labels. Species annotation was performed based on the Silva database (version 138), using the RDP classifier (version 2.2) with a 0.8 confidence threshold. The microbiome, including an estimation of alpha and beta diversities, was analyzed using the R package Vegan (version 2.5.3) for multivariate statistical analysis. The Linear discriminant analysis Effect Size (LEfSe) tool was used to determine each group’s unique cecal microbiota, with a linear discriminant analysis (LDA) score greater than 4.0 indicating unique enrichment in a treatment group [14]. Bioinformatics analyses were performed as in our previous study [13]. Microbial network construction and network topology characteristic analysis were carried out according to the method of Wen et al. [15]. The raw sequence data are available in the NCBI Sequence Read Archive (SRA) database (No. PRJNA551792).

### 2.5. Determination of Cecal Metabolites

The pre-treatment, extraction, and derivatization of cecal contents were carried out using the methodology of Li et al. [13]. Quality control (QC) samples were prepared and evaporated using a vacuum concentrator. The combined samples were then incubated with methoxyamine hydrochloride and derivatized with BSTFA. These samples were then analyzed via LC/MS (liquid chromatography/mass spectrometry) (Thermo, Ultimate 3000 LC, Q Exactive, Waltham, MA, USA) using the instrument parameter settings as detailed in Zhang et al. [16]. Metabolite annotation and data preprocessing were performed on the Majorbio cloud platform (https://www.majorbio.com/, accessed on 8 July 2023), including unsupervised principal component analysis (PCA) and partial least squares discriminant analysis (PLS-DA). Multivariate statistical analysis, using VIP (variable importance in projection) scores in PLS-DA, combined with univariate statistical analysis (Student’s *t*-test) were used to identify differentially expressed metabolites (VIP > 1 and *p* < 0.05). log2FoldChange was used to determine the extent of the upregulation or downregulation of differential metabolites in each group. The metabolic pathways associated with differential metabolites were identified using public databases, and the signal transduction pathways and biochemical metabolic pathways associated with differential metabolites were identified through KEGG pathway enrichment analysis and MetaboAnalyst 4.0 (http://www.metaboanalyst.ca/, accessed on 8 July 2023).

### 2.6. Statistical Analyses

The impacts of fermented cassava leaf diet on various growth performance metrics, intestinal morphology, and the abundance of cecal microbes in geese were assessed using one-way analysis of variance (ANOVA) in SAS v. 9.3 (SAS Institute Inc., Cary, NC, USA). All data are shown as means with standard deviations in figures. Statistically significant differences between treatments were assessed using Duncan’s multiple range tests. Significant differences between treatments was determined at *p* < 0.05 and are shown as different lowercase letters.

## 3. Results

### 3.1. Effects of Fermented Cassava Leaves on Growth Performance

**Figure 1:**
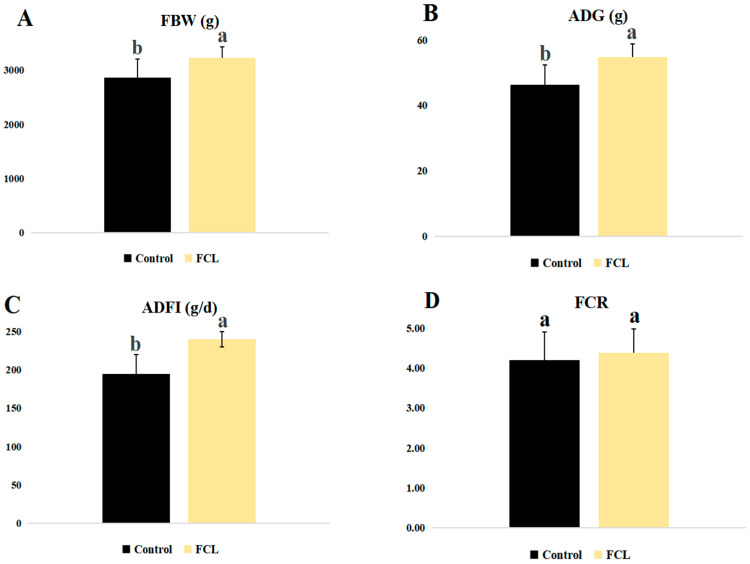
Effects of fermented cassava leaves on growth performance metrics of geese. (**A**) FBW, final body weight; (**B**) ADG, average daily weight gain; (**C**) ADFI, average daily feed intake; (**D**) FCR, feed conversion ratio. Each bar represents mean ± SD. Different lowercase letters meant significant differences (*p* < 0.05).

Geese fed with the fermented cassava leaf diet (FCL) showed increases in several growth metrics, including FBW, ADG, and ADFI (*p* < 0.05, Figure 1), as compared to control geese (CON). FCR, on the other hand, was not affected by the FCL diet (*p* > 0.05). No geese in either group died during the experiment, so the effect of the FCL diet on goose mortality could not be assessed.

### 3.2. Effects of Fermented Cassava Leaves on Intestinal Morphology

**Figure 2:**
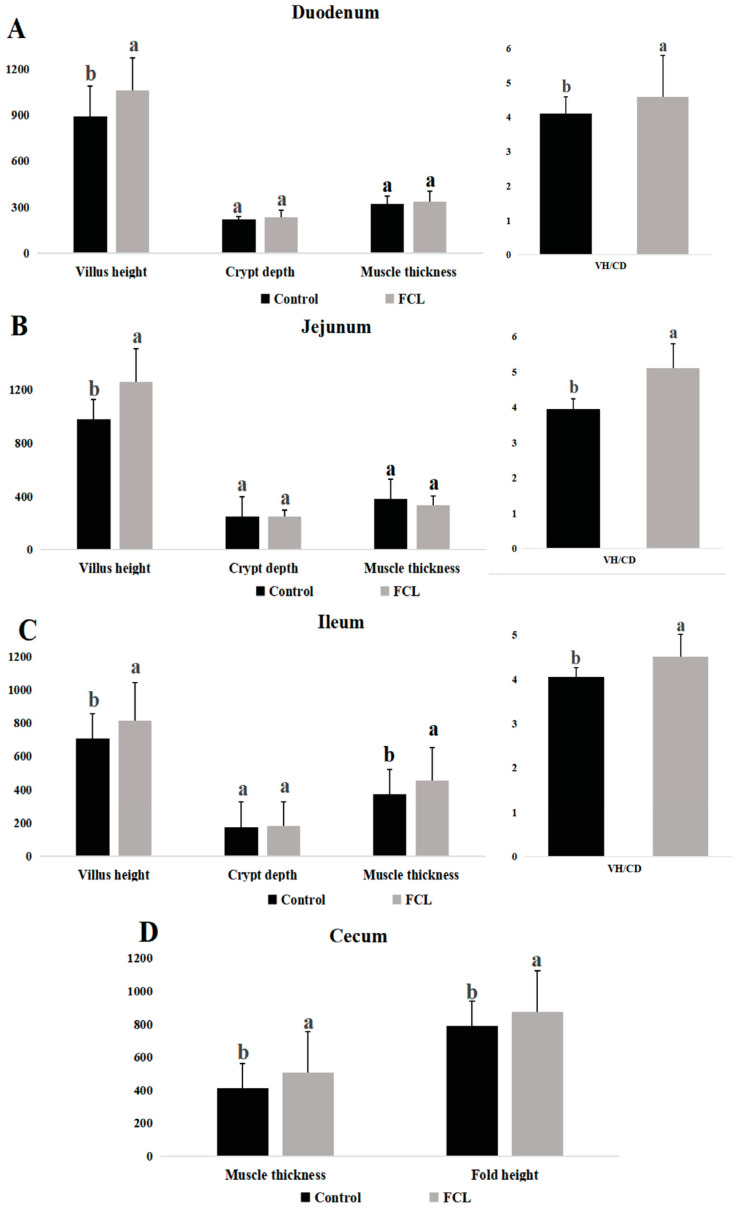
Effects of fermented cassava leaves on intestinal morphology of geese. (**A**). Villus height (VH), crypt depth (CD), muscle thickness, and VH/CD of the duodenums; (**B**) villus height (VH), crypt depth (CD), muscle thickness, and VH/CD of the jejunums; (**C**) villus height (VH), crypt depth (CD), muscle thickness, and VH/CD of the ilea; (**D**) muscle thickness and fold height of the ceca. Each bar represents mean ± SD. Different lowercase letters meant significant differences (*p* < 0.05).

The intestinal morphologies of geese in the FCL and CON groups differed in several ways (Figure 2). Geese in the FCL group had higher VH and VH/CD in their duodenums, jejunums, and ilea than CON group geese (*p* < 0.05). There were no significant differences in CD and muscle thickness in these tissues, however, between the FCL and CON geese (*p* > 0.05). Geese in the FCL group had elevated cecal muscle thickness and fold height relative to CON group geese (*p* < 0.05).

### 3.3. Effects of Fermented Cassava Leaves on the Cecal Microbiome

**Figure 3:**
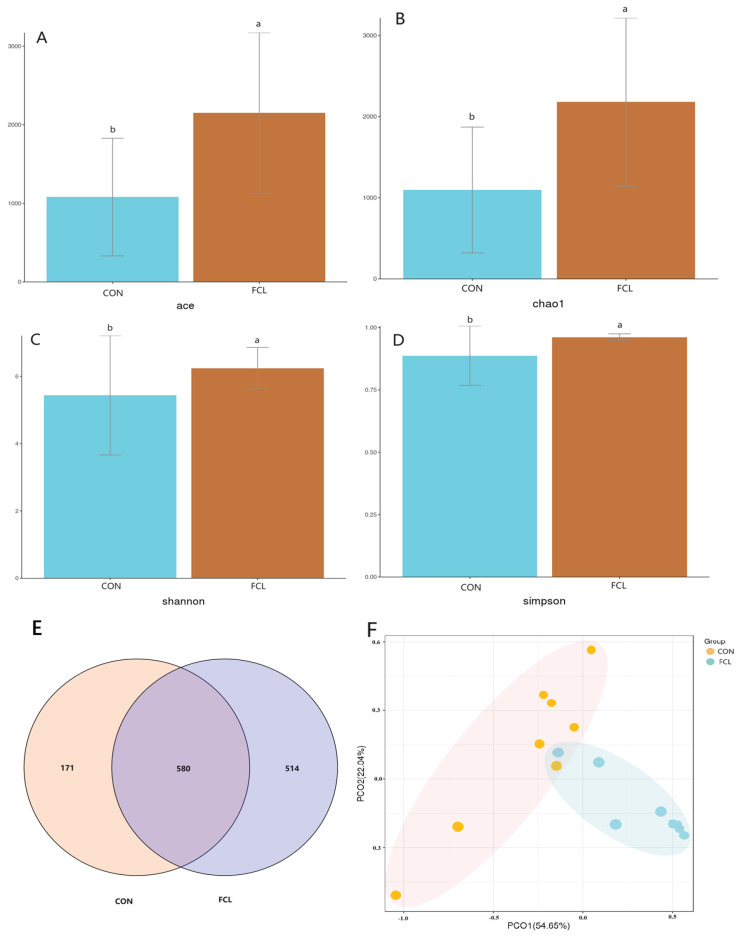
Effects of fermented cassava leaves on alpha and beta diversities in the cecal microbiota of geese. (**A**) ACE, (**B**) Chao1, (**C**) Shannon, and (**D**) Simpson indices; (**E**) Venn diagram of cecal microbes identified between FCL and CON geese; (**F**) PCoA analysis of cecal microbiota. Each bar represents mean ± SD. Different lowercase letters meant significant differences (*p* < 0.05).

Supplementation of the diet with fermented cassava leaves resulted in significant effects on cecal microbiota alpha diversity (Figure 3A–D). Microbial alpha diversity was significantly elevated in the FCL group, as measured using the ACE, Chao1, Shannon, and Simpson indices (*p* < 0.05). Venn diagram analysis revealed 580 ± 18 common OTUs between the CON and FCL groups, with 171 ± 14 OTUs unique to the CON group and 514 ± 21 OTUs unique to the FCL group (Figure 3E). These results suggested that diets with fermented cassava leaves can enhance the alpha diversity of goose cecal microbiota. The analysis of beta diversity likewise showed differences between the cecal microbiota of the two dietary treatments, with the cecal microbiota from each group clustering distinctly in the PCoA plot (Figure 3F). These results indicated that the microbial community structures within the geese ceca were significantly impacted by diets with fermented cassava leaves.

**Figure 4:**
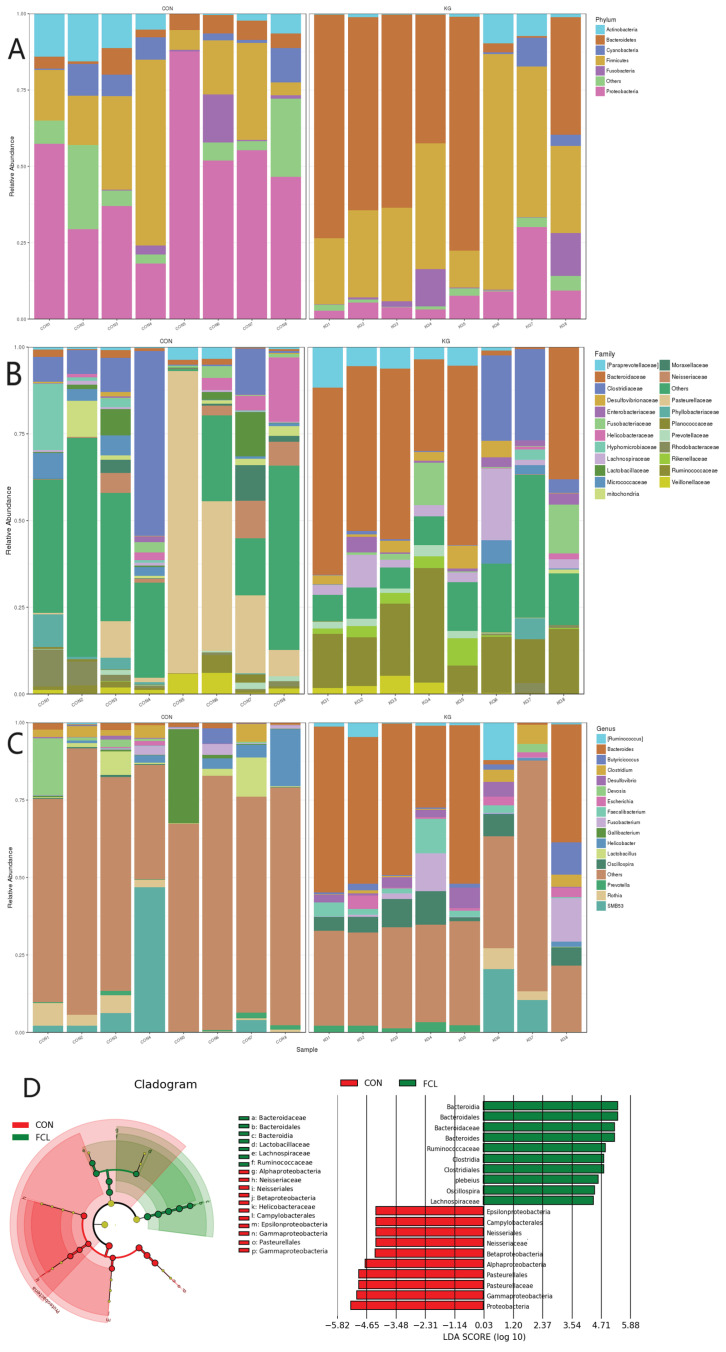
Effects of fermented cassava leaves on cecal microbial communities in geese at the (**A**) phylum, (**B**) family, and (**C**) genus levels; (**D**) Linear discriminant analysis Effect Size (LEfSe) analysis of cecal microbial communities of the two treatment groups.

The cecal microbial community structures of geese treated with different diets are shown in Figure 4. In CON geese, the most abundant microbes at the phylum level (Figure 4A) were Proteobacteria (47.92% ± 4.93%), Firmicutes (23.06% ± 5.15%), Actinobacteria (6.93% ± 1.10%), Cyanobacteria (4.93% ± 0.93%), Bacteroidetes (4.8% ± 0.44%), and Fusobacteria (2.63% ± 0.26%). For the FCL group, they were Bacteroidetes (45.09% ± 6.77%), Firmicutes (36.14% ± 4.99%), Proteobacteria (8.88% ± 2.97%), Fusobacteria (3.7% ± 0.87%), and Actinobacteria (2.64% ± 0.7%). The most abundant microbes in the CON group at the family level (Figure 4B) consisted primarily of *Pasteurellaceae* (21.53% ± 3.12%), *Clostridiaceae* (11.45% ± 1.19%), *Helicobacteraceae* (3.68% ± 0.66%), *Neisseriaceae* (3.48% ± 0.81%), *Lactobacillaceae* (3.11% ± 0.37%), and *Hypomicrobiaceae* (2.91% ± 0.62%). The FCL group consisted of *Bacteroidaceae* (33.60% ± 4.64%), *Ruminococcaceae* (15.82% ± 4.91%), *Clostridiaceae* (7.12% ± 1.26%), *Lachnospiraceae* (5.66% ± 1.34%), *Paraprevotellaceae* (4.17% ± 0.43%) *Fusobacteriaceae* (3.70% ± 0.39%), and *Desulfovibrio* (2.57% ± 0.59%). The main microorganisms identified in the CON group geese at the genus level (Figure 4C) were *Gallibacter* (4.10% ± 0.62), *Helicobacter* (3.66% ± 0.18%), *Lactobacillus* (3.11% ± 0.56%), and *Devosia* (2.81% ± 0.22%). The FCL group mainly consisted of *Bacteroides* (33.43% ± 5.71%), *Oscillospira* (5.48% ± 0.67%), *Fusobacterium* (3.7% ± 0.84%), and *Faecalibacterium* (2.93% ± 0.59%). In addition, LEfSe (Linear discriminant analysis Effect Size) was used to identify the most differentially abundant microbes between the CON and FCL groups (Figure 4D). The indicator microorganisms in the FCL group were *Bacteroidia* and its constitutive taxa (*Bacteroidales*, *Bacteroidaceae*, and *Bacteroides*), *Ruminococcaceae*, *Clostridia* (*Clostridiales*), *Oscillospira*, and *Lachnospiraceae*. The microorganisms enriched in the CON group were Proteobacteria (*Gammaproteobacteria*, *Alphaproteobacteria*, and *Betaproteobacteria*), *Pasteurellaceae* (*Pasteurellales*), *Gallibacterium*, *Neisseriaceae* (*Neisseriales*), and *Campylobacterales*.

**Figure 5:**
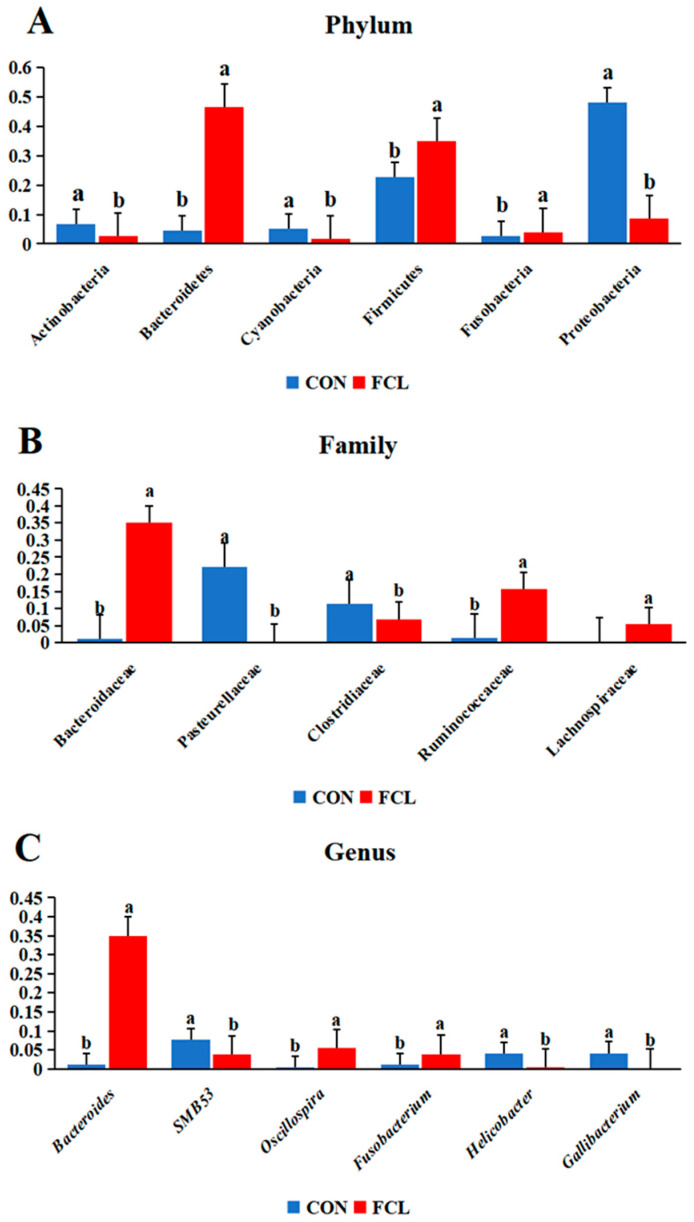
Comparative analysis of differential abundances of cecal microbes in geese treated with different diets at the (**A**) phylum, (**B**) family, and (**C**) genus levels. Each bar represents mean ± SD. Different lowercase letters mean significant differences (*p* < 0.05).

The cecal microbiota differed significantly between geese fed with different diets (Figure 5). In the FCL group, the phyla Bacteroidetes and Firmicutes were significantly more abundant (*p* < 0.05) than in the CON group, though Proteobacteria, Actinobacteria, and Cyanobacteria were significantly less abundant (*p* < 0.05). At the family level, *Ruminococcaceae*, *Bacteroidaceae*, and *Lachnospiraceae* were more abundant in the FCL group (*p* < 0.05), while *Pasteurellaceae* and *Clostridiaceae* were more abundant in the CON group (*p* < 0.05). We also found that the feeding of diets supplemented with fermented cassava leaves significantly increased the abundances of the genera *Bacteroides* and *Oscillospira* in the cecal microbiota.

**Figure 6:**
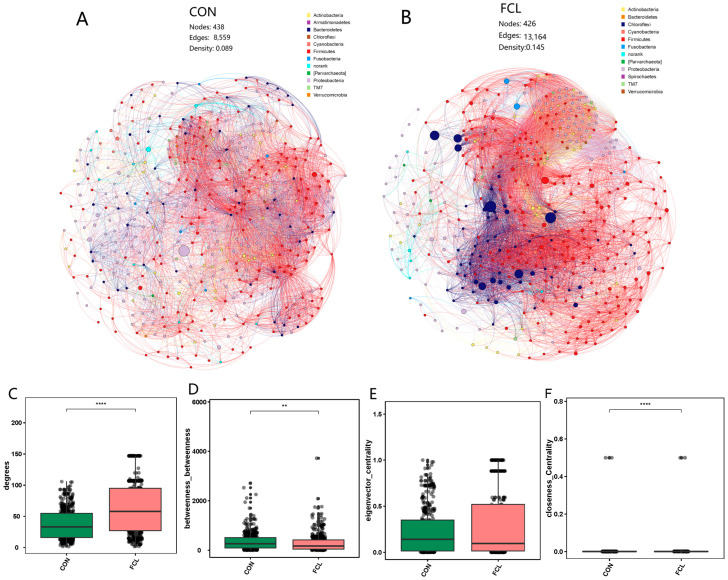
Co-occurrence network analysis of cecal microbial communities of geese treated with different diets. (**A**) Network of CON; (**B**) network of FCL; (**C**) degree; (**D**) betweenness; (**E**) eigenvector centrality; (**F**) closeness centrality. Each bar represents mean ± SD. ** Represents significant difference (*p* < 0.01); **** represents significant difference (*p* < 0.0001).

Co-occurrence networks of the cecal microbiota of geese fed with different diets were structured (Figure 6A,B). At the network level, the two groups had similar node numbers (438 ± 21 versus 426 ± 13), and the FCL group (13,164 ± 239 edges, 0.145 ± 0.032 density) had more edges and a higher density (*p* < 0.05) than the CON group (8559 ± 168 edges, 0.089 ± 0.011 density). These results demonstrated that the links and interactions among microbial communities in the FCL group was more complex. At the node level, the network topological characteristics of the two groups were compared. We found that the degree was higher (*p* < 0.0001) for FCL than for CON (Figure 6C). This trend implies that the bacterial taxa in the network of FCL were more located in the central positions than those in CON. Meanwhile, the value of closeness centrality in FCL was also significantly higher than that in CON (Figure 6F), indicating that the nodes in the network of FCL were closer than those in CON (*p* < 0.0001). The eigenvector centrality of the two groups did not have any obvious differences, and it is possible that the importance of nodes in the two networks is similar (Figure 6E). However, the betweenness of FCL was significantly lower than that of CON (*p* < 0.01). This means that there are more connection nodes in the CON network than in the FCL group (Figure 6D).

### 3.4. Effects of Fermented Cassava Leaves on the Cecal Metabolome

**Figure 7:**
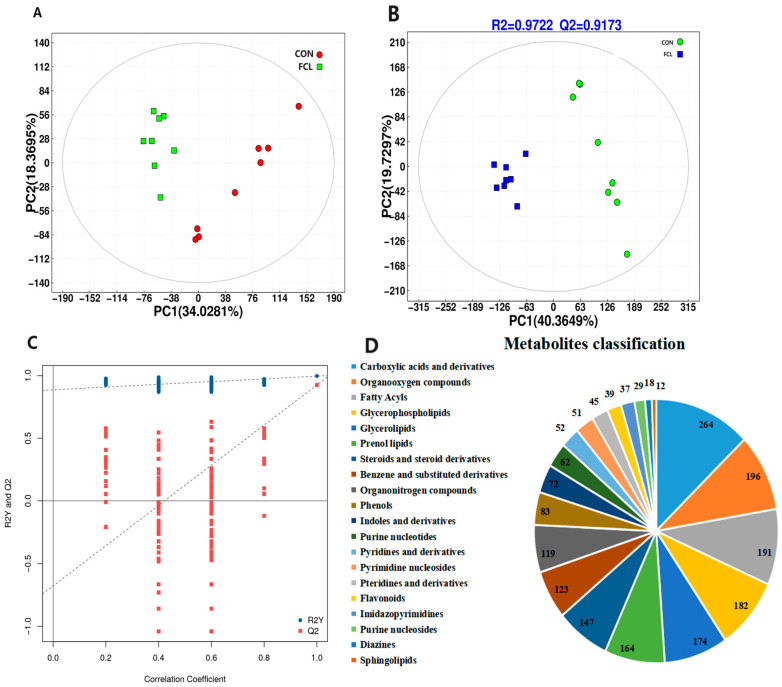
Effects of fermented cassava leaves on cecal metabolites in geese. (**A**) PCA of goose cecal metabolites; (**B**) PLS-DA analysis of goose cecal metabolites; (**C**) permutation validation testing of PLS-DA; (**D**) classification (top 20) of goose cecal metabolites.

After filtering, the analysis of the cecal metabolome revealed 14,465 high-quality ions and 6526 metabolites. Principal component analysis (PCA) indicated clear differences in the cecal metabolites of geese fed the control diet and the fermented cassava leaf diet (Figure 7A). Partial least squares discriminant analysis (PLS-DA) was used to detect the most discriminating metabolites between the two treatment groups. The values of R^2^Y and Q^2^ in the present study were 0.9722 and 0.9173. These high values, both higher than 0.80, illustrated the stability and reliability of the model (Figure 7B). These results indicated that the distributions of cecal metabolites in the FCL group and the CON group differed significantly. To further examine the reliability of the PLS-DA model, we also performed permutation validation testing (Figure 7C). The positive slope of the Q2Y regression line indicated that the model was reliable. Moreover, the R2Y values (blue dots) were observed to be higher than the Q2 values (red dots), indicating independence between the training and testing sets. Of the 6526 metabolites identified in the goose ceca, the most common classes were carboxylic acids and derivatives, organooxygen compounds, fatty acyls, glycerophospholipids, glycerolipids, and prenol lipids (Figure 7D).

**Figure 8:**
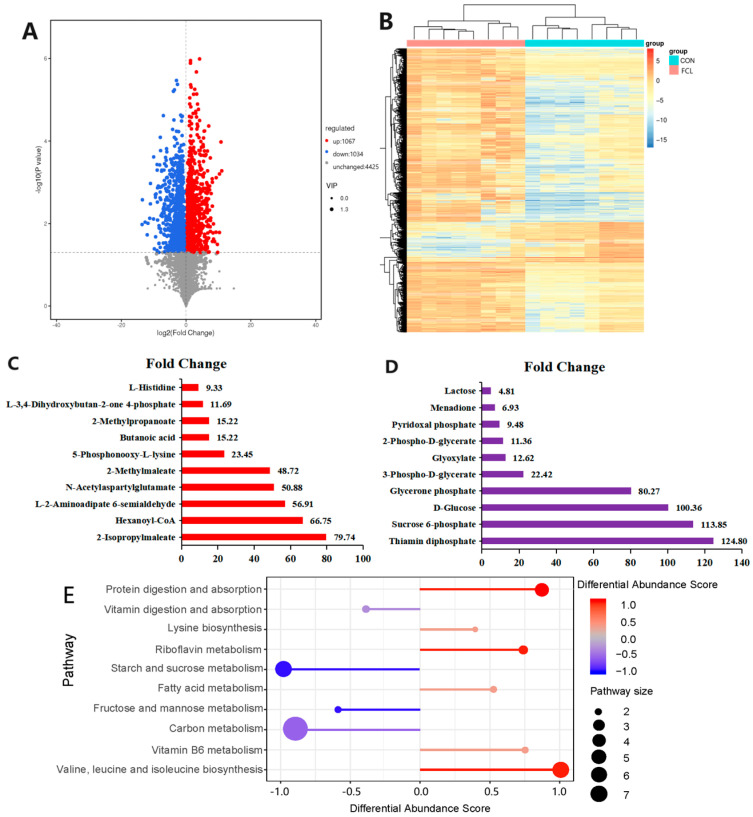
Effects of fermented cassava leaves on cecal metabolites and associated metabolic pathways in geese. (**A**) Volcano plot analysis of goose cecal metabolites (CON vs. FCL); (**B**) differential metabolite clustering heatmap; (**C**) upregulated metabolites with top 10 largest fold change (FC) values; (**D**) downregulated metabolites with top 10 largest fold change (FC) values; (**E**) differential abundance score analysis of metabolic pathways enriched among differential metabolites (CON vs. FCL).

Changes in metabolite levels between the CON group and FCL group were visualized using a volcano plot (Figure 8A). A total of 2101 differential metabolites were identified in this study, with 1067 upregulated and 1034 downregulated metabolites in the FCL group. Using cluster heatmap analysis, the differential metabolites from the two dietary treatments were found to cluster distinctly (Figure 8B), implying a close relationship between diet and cecal metabolites.

To further clarify the effects of the fermented cassava leaf diet on cecal metabolites in geese, we identified the top ten upregulated and downregulated metabolites based on the significance of the differences in fold change values. Upregulated metabolites in the FCL group included 2-isopropylmaleate, hexanoyl-CoA, l-2-aminoadipate 6-semialdehyde, N-acetylaspartylglutamate, 2-methylmaleate, 5-phosphonooxy-l-lysine, butanoic acid, 2-methylpropanoate, l-3,4-dihydroxybutan-2-one 4-phosphate, and l-histidine (Figure 8C). Meanwhile, downregulated metabolites in the FCL group included thiamin diphosphate, sucrose 6-phosphate, d-glucose, glycerone phosphate, 3-phospho-d-glycerate, glyoxylate, 2-phospho-d-glycerate, pyridoxal phosphate, menadione, and lactose (Figure 8D).

In order to gain a deeper understanding of how fermented cassava leaves affected cecal metabolism, we performed metabolic pathway enrichment analysis on the set of differentially abundant metabolites. Using the differential abundance scores (Figure 8E), differential metabolites upregulated in the CON group were enriched for vitamin digestion and absorption, carbon metabolism, fructose and mannose metabolism, and starch and sucrose metabolism pathways. Protein digestion and absorption, lysine biosynthesis, valine, leucine and isoleucine biosynthesis, riboflavin metabolism, fatty acid metabolism, and vitamin B6 metabolism pathways were enriched among upregulated differential metabolites in the FCL group. These enriched metabolic pathways are known to be involved in fatty acid metabolism, amino acid synthesis metabolism, vitamin synthesis metabolism, and carbohydrate digestion, indicating that the supplementation of feed with fermented cassava leaves alters the metabolic pathways of nutrients and functional components in the goose cecum.

## 4. Discussion

Fiber-rich forage has been widely used in the poultry farming industry, as fiber can provide the host with the needed energy and promote the development of the digestive tract, thus boosting animal growth performance [2,17]. Compared with other poultry, geese have more developed gizzards and ceca and possess an excellent ability to digest and utilize fibers [3,5].

There have been many reports on the benefits of feeding fermented forage on growth performance in geese. Li et al. [2] showed that feed supplemented with fermented alfalfa-mixed silage noticeably enhanced the growth performance of Lande geese. Fermented maize stover could also be used to replace up to 15% of concentrated feed, with improvements in production performance [3]. Another study reported that geese fed with a paper mulberry silage diet also showed increases in ADG [4]. The results detailed here were in agreement with these previous studies. Unlike the studies that illustrated the positive effects of fermented feed on growth performance, feeding forage without fermented material has been found to produce mixed results. Jin et al. [18] found that feeding a *Pennisetum hydridum* diet significantly improved the BW, ADG, and ADFI of geese. However, geese fed with fresh alfalfa showed no improvements in growth performance [17]. In addition to taxon-specific differences in forage, whether forage is fermented also impacts its quality as feed material. The fermentation of forage can alter its physical structure, improve its palatability, and increase the contents of organic acids, active enzymes, functional metabolites, and probiotics such as lactic acid bacteria [6]. These advantages can explain to some extent why feeding fermented forage can improve growth performance, though its mechanism of action needs further clarification.

In monogastric animals, the intestine is vital for the digestion and absorption of nutrients [3,5,6]. Metrics of intestinal morphology, including VH, CD, VH/CD, and muscle thickness, are valuable indicators of the digestive and absorptive functions of the intestine [6]. Intestinal villi are the primary structures that absorb nutrients. When VH increases, the intestine’s capacity to absorb nutrients also increases. CD can reflect the renewal rate of intestinal epithelial cells. An increase in VH/CD indicates a larger intestinal endometrial area, improved intestinal mucosal structure, and increased villus cell density, thus enhancing digestive and absorptive functions [19]. In this study, both the VH and VH/CD of each intestinal segment and the cecal muscle thickness and fold height were significantly increased in geese fed with fermented cassava leaves. This indicated that feeding geese with fermented cassava leaves can improve the morphology and structure of the intestinal mucosa. Some previous studies have reported similar phenomena. Geese fed with alfalfa-mixed silage, fermented maize stover, and fermented feed all had enhanced intestinal morphology indexes, which are beneficial for feed digestion, and increased fiber, supporting the promotion of growth performance [2,3,6].

Microorganisms in the gut also play significant roles in the digestion and utilization of dietary fiber in geese [6,7]. In this study, we observed higher alpha diversity indicators and OTU numbers in the ceca of geese fed with fermented cassava leaves. This differed from other studies on fermented forage feed, as the alpha diversity of the cecal microbiota in geese was not found to be impacted by fermented maize stover or other fermented feeds [3,6]. However, these investigations consistently found that feeding fermented forage can significantly alter the beta diversity of the cecal microbiota in geese, indicating that the composition of feed can alter the community structure of microorganisms [3,6].

*Bacteroidetes* are commonly found in the guts of various animals, playing important roles in the digestion and utilization of complex polysaccharides, starch, and cellulose, decomposing and converting them into simpler compounds [20,21]. Several reports have identified *Bacteroidetes* as some of the most abundant bacteria in goose ceca. Deng et al. [7] and Fang et al. [19] both identified *Bacteroidetes* as the most abundant bacterial genus in goose ceca, with similar abundances to our previous report [10]. Yan et al. [6] also demonstrated that fermented feeds could increase the abundance of *Bacteroidetes* in goose intestines, promoting their adaptation to different diets and the digestibility of nutrients. This study produced similar results, showing that a diet with fermented cassava leaves improved digestion and the utilization of nutrients. However, the dominant microorganisms in the ceca of geese identified here did differ from those identified in other studies. For instance, Hong et al. [3] found that fermented maize stover resulted in increased abundances of beneficial bacteria (*Coprococcus* and *Victivallis*) in the goose gut. Yu Jun et al. [1] and Deng et al. [7] observed that *Alistipes* and *Clostridia*_UCG-014 were the most abundant species in the ceca of geese fed with cottonseed meal or grape seed procyanidins. These phenomena indicate that dietary composition can impact the make-up of the cecal microbiota in geese, and thus it is necessary to conduct further research on gut microbial diversity and how it impacts the utilization of different feed sources.

To investigate the interactions between the gut microbiota, co-occurrence network analysis was applied in some recent animal studies which discovered significant differences in network characteristics among different gut microbiota clusters [1]. Liu et al. [22] reported that the gut microbiota networks of small domestic ruminants fed high-protein diets were more complex than those of wild ruminants fed low-protein diets, as reflected in a higher number of nodes, edges, and modules. Similar reports have also been made in poultry research. Hu et al. [23] observed the topological parameters of co-occurrence networks and found a simpler network pattern in the cecal microbiota of laying hens fed a low-energy and low-protein diet. Consistent with the above studies, our study found that feeding geese with fermented cassava leaf feed could enhance the complexity of the cecal microbiota network, which may be related to the high protein content of cassava leaves promoting gut microbiota interactions.

This study applied metabolomics techniques to analyze metabolite contents in the ceca of geese fed different diets. Feeding fermented cassava leaves resulted in significant changes in the presence and abundance of metabolites, which are of great importance for understanding the mechanisms by which cassava leaf diets affect animal health and growth performance. Metabolites upregulated in the FCL group included those involved in amino acid biosynthesis, protein digestion and absorption, riboflavin metabolism, and fatty acid metabolism. 2-isopropylmaleate is a substrate for isopropylmalate isomerase, which regulates the biosynthesis of isoleucine and valine [24,25]. L-2-aminoadipate 6-semialdehyde is believed to function in the biosynthesis and degradation of lysine [26]. N-acetylaspartylglutamate functions in the synthesis and degradation of alanine, aspartate, and glutamate through the metabolic pathway [27,28]. Hexanoyl-CoA is the precursor of short-chain fatty acyl-coenzyme A (CoA), which is an important intermediate for fatty acid synthesis [29,30]. 2-methylmaleate is involved in the catalytic process of producing malic acid, which is an important intermediate product that is easily absorbed by the body, making it an excellent food additive and functional food [31,32]. Butanoic acid is involved in the digestion and absorption of carbohydrates and proteins and has a promoting effect on animal intestines and health [16,33]. In the CON group, we found significant increases in metabolites involved in carbohydrate catabolism, such as thiamin diphosphate. This metabolite, which is the active form of thiamine, functions as a cofactor for multiple enzymes involved in carbohydrate catabolism [34]. Several other intermediates of carbohydrate metabolism also increased in the CON group, including sucrose 6-phosphate, d-glucose, glycerone phosphate, 3-phospho-d-glycerate, glyoxylate, 2-phospho-d-glycerate, menadione, and lactose [35]. Pyridoxal phosphate, a metabolite considered to be vital to vitamin B6 metabolism and vitamin digestion and absorption [36], was also more abundant in the CON group.

Previous research has suggested that diet can alter the composition of gut metabolites and associated metabolic pathways. Deng et al. [7] showed that goose diets supplemented with antioxidant grape seed procyanidins resulted in an upregulation of dinarginine, proline, and glutathione and a downregulation of spermine and N-acetylputrescine. This diet also promoted amino acid metabolism pathways and inhibited inflammation-related metabolic pathways. Inulin is a plant polysaccharide that can promote the growth of gut probiotics. In dairy cows, inulin has been shown to impact rumen metabolites and metabolic pathways, especially those related to amino acids, vitamins, and secondary metabolites [36]. Moreover, we previously found in a study of Hainan black goats that cassava foliage silage resulted in an upregulation of metabolites associated with protein digestion and absorption and the metabolism of both nucleotide sugars and amino sugars [13]. In this study, changes in the composition and abundance of vitamins, amino acids, sugars, and digestive and absorptive intermediates in the ceca of geese fed with fermented cassava leaves were also consistent with changes in the expression of metabolic pathways. However, there has still been relatively little metabolomics research on the intestines of geese, and few metabolites have been reported. Thus, it is critical to further explore the metabolic regulation of host genetics, reproduction, nutrition, immunity, and stress in geese.

## 5. Conclusions

Diet supplemented with FCL was beneficial to growth performance in geese, resulting in higher FBW, ADG, and ADFI. The FCL diet could also promote a healthier intestinal morphology, as reflected by greater VH, VH/CD, and cecal fold height. The FCL diet also resulted in significant changes in microbiota diversity, composition, and interaction in the cecum, increasing the diversity and the abundance of the beneficial *Bacteroides*. It likewise enhanced the complexity and stability of the microbial co-occurrence network. Furthermore, the FCL diet affected the composition and abundance of cecal metabolites, with increases in metabolites associated with amino acid biosynthesis, digestion, and absorption and the upregulation of associated metabolic pathways. Based on the numerous positive impacts of the FCL diet on growth performance, intestinal development, and gut microbe-mediated metabolism in geese, FCL should be considered and utilized as an excellent feed resource for geese in tropical and subtropical regions.

## Figures and Tables

**Table 1:** Ingredient and nutrient composition (g/kg, as feed) of the control diet (CON) and fermented cassava leaf diet (FCL).

Items	CON	FCL	Fermented Cassava Leaves
Ingredient (%)			
Corn	59.5	55.5	
Soybean meal	20	15	
Wheat	6	7	
Wheat bran	11	12	
Fermented cassava leaves	0	7	
Limestone powder	2	2	
Calcium hydrogen phosphate	0.2	0.2	
DL-Met	0.3	0.3	
NaCl	0.2	0.2	
Premix compound ^a^	0.8	0.8	
Total	100	100	
Nutrient composition ^b^			
Metabolizable energy (MJ/kg)	11.78	11.83	
Crude protein (%)	15.72	15.63	20.45
Crude fiber (%)	5.24	5.33	18.21
Neutral detergent fiber (%)	20.55	26.18	31.24
Acid detergent fiber (%)	12.06	17.23	22.90
Calcium (%)	0.9	0.9	1
Phosphorus (%)	0.45	0.45	0.4
Lysine (%)	0.94	0.89	1
Methionine (%)	0.56	0.5	0.1
HCN (mg/kg DM)	0	1.7	24.06

^a^ The premix provided the following per kg of diet: VA 20,000 IU, VD 10,000 IU, VE 4500 mg, VK 10 mg, VB_1_ 30 mg, VB_2_ 150 mg, VB_6_ 60 mg, VB_12_ 0.5 mg, nicotinic acid 3 g, pantothenic acid 40 mg, folic acid 10 mg, biotin 1 mg, Fe 120 mg, Cu 8 mg, Zn 120 mg, Mn 2000 mg, Se 0.5 mg, I 0.8 mg. ^b^ Metabolizable energy is calculated values, and others are analyzed values.

## Data Availability

The original contributions presented in the study are included in the article, further inquiries can be directed to the corresponding author.
